# Molecular phylogeny of the antiangiogenic and neurotrophic serpin, pigment epithelium derived factor in vertebrates

**DOI:** 10.1186/1471-2164-7-248

**Published:** 2006-10-04

**Authors:** Xuming Xu, Samuel Shao-Min Zhang, Colin J Barnstable, Joyce Tombran-Tink

**Affiliations:** 1 Department of Ophthalmology and Visual Science, Yale University School of Medicine, New Haven, CT 06520, USA; 2 Department of Neural and Behavioral Sciences, Penn State University College of Medicine, Hershey, PA 17033, USA

## Abstract

**Background:**

Pigment epithelium derived factor (PEDF), a member of the serpin family, regulates cell proliferation, promotes survival of neurons, and blocks growth of new blood vessels in mammals. Defining the molecular phylogeny of PEDF by bioinformatic analysis is one approach to understanding the link between its gene structure and its function in these biological processes.

**Results:**

From a comprehensive search of available DNA databases we identified a single PEDF gene in all vertebrate species examined. These included four mammalian and six non-mammalian vertebrate species in which PEDF had not previously been described. A five gene cluster around PEDF was found in an approximate 100 kb region in mammals, birds, and amphibians. In ray-finned fish these genes are scattered over three chromosomes although only one PEDF gene was consistently found. The PEDF gene is absent in invertebrates including Drosophila melanogaster (*D. melanogaster*), Caenorhabditis elegans (*C. elegans*), and sea squirt (*C. intestinalis*). The PEDF gene is transcribed in all vertebrate phyla, suggesting it is biologically active throughout vertebrate evolution. The multiple actions of PEDF are likely conserved in evolution since it has the same gene structure across phyla, although the size of the gene ranges from 48.3 kb in *X. tropicalis *to 2.9 kb in fugu, with human PEDF at a size of 15.6 kb. A strong similarity in the proximal 200 bp of the PEDF promoter in mammals suggests the existence of a possible regulatory region across phyla. Using a non-synonymous/synonymous substitution rate ratio we show that mammalian and fish PEDFs have similar ratios of <0.13, reflecting a strong purifying selection of PEDF gene. A large number of repetitive transposable elements of the SINE and LINE class were found with random distribution in both the promoter and introns of mammalian PEDF.

**Conclusion:**

The PEDF gene first appears in vertebrates and our studies suggest that the regulation and biological actions of this gene are preserved across vertebrates. This comprehensive analysis of the PEDF gene across phyla provides new information that will aid further characterization of common functional motifs of this serpin in biological processes.

## Background

The large supergene family of serpins encodes two groups of proteins. One group comprises the predominant family of protease inhibitors in mammals and regulates cascades such as inflammation, blood coagulation and extracellular matrix remodeling. The second group represents a substantial number of serpins that, though structurally similar, are not thought to be inhibitors of specific proteases but have important functions in a wide range of cellular processes.

Pigment Epithelium Derived Factor (PEDF) is a member of the second group of serpins. It is not known to target any specific protease but is associated with two important cellular processes: proliferation and survival. By shifting the balance between proliferation and differentiation, PEDF slows the expansion of tumor cells [1-3]. In cultured fibroblasts PEDF promotes exit from the cell cycle and movement into a GO phase [4]. PEDF also inhibits the growth of endothelial cells that form new blood vessels in cancers and in some neovascular diseases, particularly in the eye [1-3,5-7]. More recently PEDF has been shown to promote self-renewal of adult stem cells [8].

In addition to its effects on proliferation, PEDF increases survival of neurons. It interferes with progression of neurodegenerative cascades promoted by axotomy, glutamate excitotoxicity, and oxidative stress in many types of neurons (reviewed in [9]). Similarly, this serpin protects vertebrate photoreceptor cells from damage induced by removal of trophic support [10,11] or exposure to chronic bright light [12].

The multiple biological roles of this serpin have been confirmed in PEDF null mice that show epithelial hyperplasia, increased endothelial cell proliferation, and increased microvessel density in several organs [13]. We have previously identified potentially important functional domains in the PEDF molecule, several of which have been tested in both in vitro and in vivo experimental models [14-16].

PEDF is a 50 kDa secreted protein and is widely distributed in human tissues [17-19]. Characterization of the human gene for PEDF [19,20] paved the way for the derivation of its crystal structure at a resolution of 2.85Å [21].

Serpins have been organized into 16 clades based on the sequence similarities of the proteins and exon-intron organization of the genes [22,23]. PEDF falls into the serpin Fl clade based on the few mammalian sequences available. In this study, we have used computational tools to identify PEDF, and other serpin, genes in a variety of additional species. Comparison of the structure of the PEDF gene, and the surrounding genes, in various species has extended our early work on the evolutionary conservation and genomic structure of PEDF [19]. Using computational tools we have identified PEDF genes in 10 additional species and 20 additional serpin genes. We have confirmed that many of these additional PEDF sequences are expressed by RT-PCR. These new sequences have allowed the refinement of the serpin phylogenetic tree and show the relatedness of PEDF to other serpins.

## Results

### PEDF coding sequences is present only in the vertebrates

PEDF sequences have previously been described from human (*Homo sapiens*), mouse (*Mus musculus*), cow (*Bos taurus*), and rat (*Rattus norvegicus*). We obtained the PEDF coding sequences for chimpanzee (*Pan troglodytes*, [GenBank:BK005454]), chicken (*Gallus gallus*, [GenBank:BK005453]) and fugu (*Fugu rubripes*, [GenBank:BK005455]) from genomic databases, and have used other databases to identify or assemble the coding sequences for medaka (*Oryzias latipes*, [GenBank:BK005456]), pig (*Sus scrofa*, [GenBank:BK005457]), trout (*Oncorhynchus mykiss*, [GenBank:BK005458]), African clawed frog (*Xenopus laevis*, [GenBank:BK005459]), western clawed frog (*Xenopus tropicalis*, [GenBank:BK005463]), zebrafish (*Danio rerio*, [GenBank:BK005460]), and dog (*Canis familiaris*, [GenBank:BK005764]) PEDF. In total we have identified the PEDF sequence in an additional three mammalian and seven non-mammalian species. We did not find a PEDF gene in the fly (*D. melanogaster*) or worm (*C. elegans*) genome databases, or in the sea squirt (*C. intestinalis*) the closest invertebrate to the vertebrate lineage.

### Serpin F2 and C1 inhibitor are the closest relatives of PEDF

As might be expected, alignments of the PEDF DNA sequences reveal few homologies. However, when these sequences are compared pairwise, the relatedness within each phylum is very clear (Table 1).

**Figure 1:**
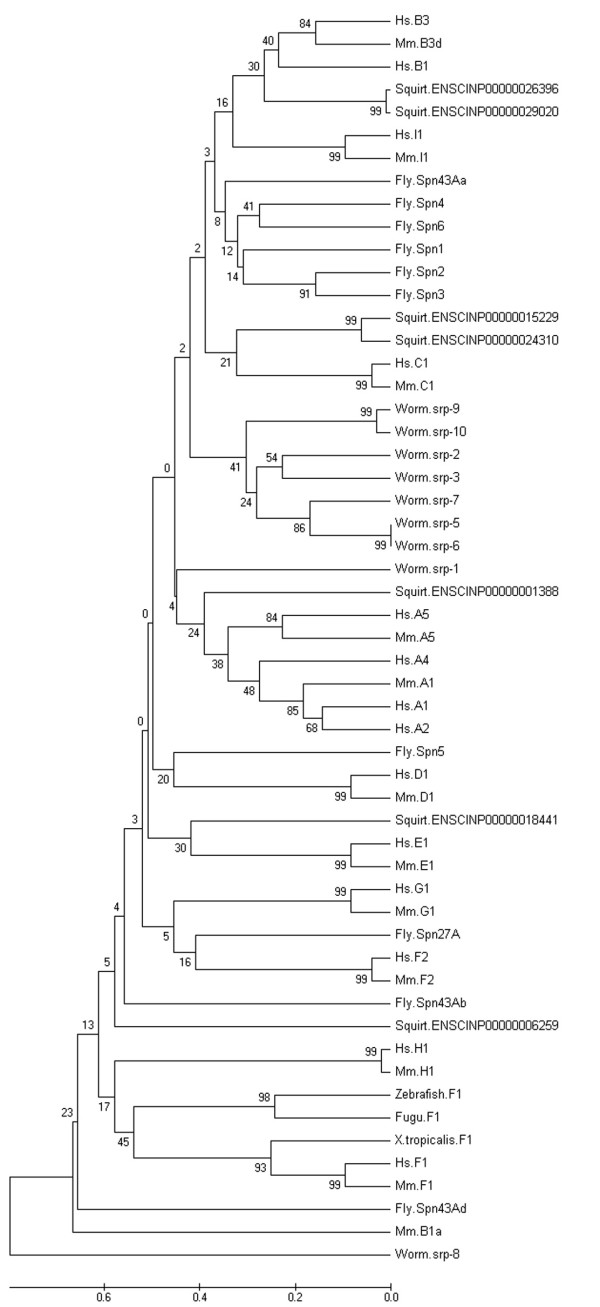
Phylogenetic relationship of serpins in selected vertebrates and invertebrates. Protein sequences of available serpins in sea squirt, fly, and worm, also of typical serpins in human and mouse were used for this analysis. Label prefixes "Hs", "Mm", "worm", "squirt" and "fly" were used for genes for human, mouse, worm, sea squirt and fly respectively. This is a bootstrap consensus tree based on 500 replicates. The numbers on nodes are bootstrap values.

The phylogenetic relationships of many serpins have been described previously [22]. As a by-product of searching for PEDF genes we identified 13 homologues of other serpins in many vertebrates, and 7 serpin homologues in sea squirt, one of the invertebrate species with closest relationship with vertebrate. Previous studies also have identified a number of serpin genes in both fly and worm. Our phylogenetic analysis shows that none of these invertebrate serpins are orthologous to the PEDF gene. A few show some relationships with other clades of human and mouse serpins (Fig. 1). The data strongly suggest that the PEDF gene first appeared in vertebrates.

**Figure 2:**
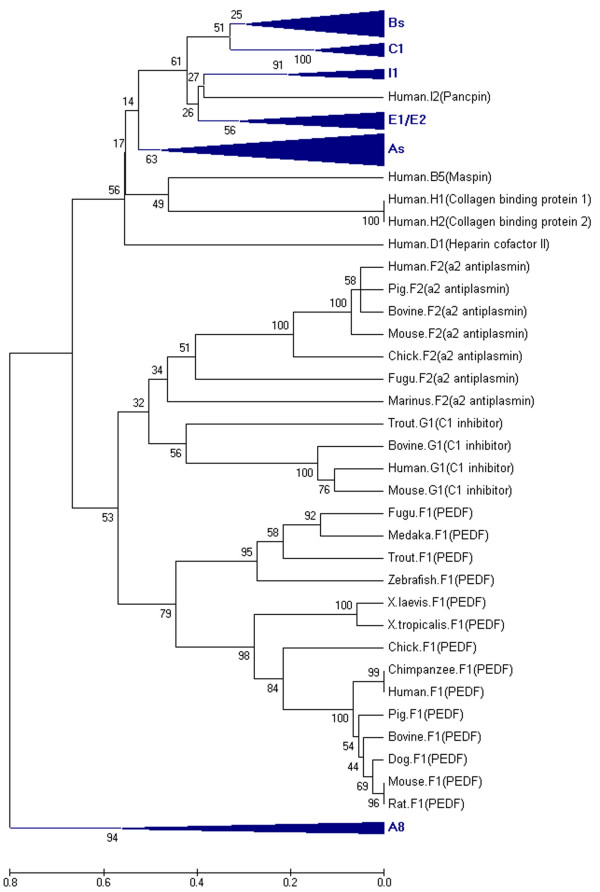
Phylogenetic relationship of PEDF to other serpins. This is a short list of Additional file 1 which include 88 serpins with 14 PEDF sequences used to construct this bootstrap consensus tree based on 500 replicates. The numbers on nodes are bootstrap values.

The phylogenetic relationships among PEDF and other vertebrate serpins are shown in Figure 2 (full list in Additional file 1). Although some parts of the phylogenetic tree are not reliable enough, with a bootstrap score less than 50, the sub-tree consisting of PEDF sequences and their close relatives are validated by most scores above 50. The PEDF sequences maintain the expected phylogenetic relationships among the species. For example, the fish and the mammalian PEDFs are both clustered appropriately within their respective phyla. In previous phylogenetic trees constructed from a smaller number of sequences, the serpin most closely related to PEDF (serpin F1, clade F1) is Serpin F2/alpha-2-antiplasmin (clade F2), which is adjacent to PEDF on chromosome 17 (Fig. 2). The next closest PEDF relative is complement C1 inhibitor (clade G1) that maps to chromosome 11ql2-q13.1. With the additional sequences we have identified, this region of the tree is now reorganized and shows that Serpin F2 and CI inhibitor appears to be more closely related to each other than to PEDF though they are still the closest relatives of PEDF.

### The PEDF gene is transcribed in many vertebrate species

**Figure 3:**
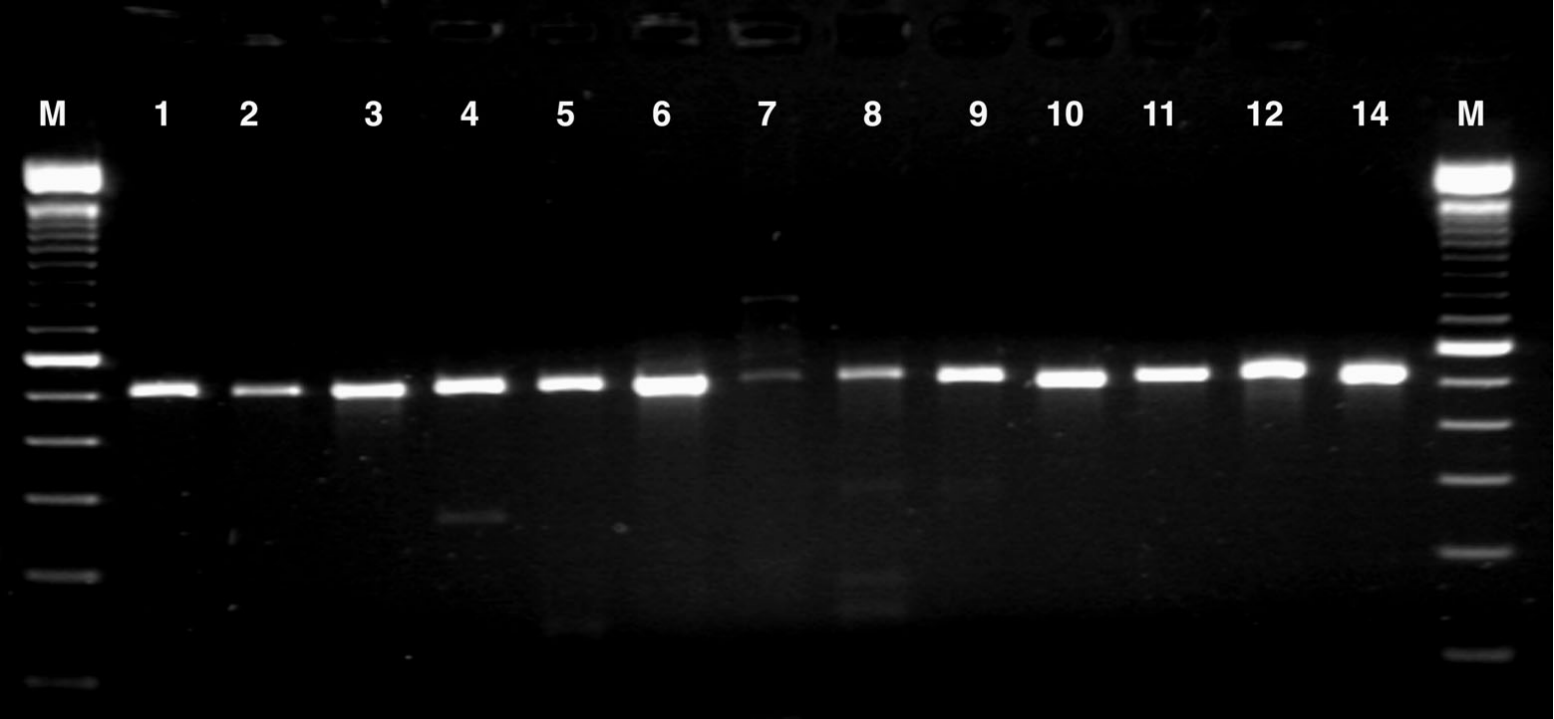
Amplification of serpin sequences to verify the expression of genes identified in databases. RNA and cDNA were prepared from brain or cell lines from the species listed and amplified using primers listed in Table 4. All primer pairs correspond to different exons ensuring that amplicons arise from mature RNA. Lanes 1, 100 bp marker; 2, Frog E2; 3, Frog F1; 4, Zebrafish F1; 5, Chick F1; 6, Fugu F1; 7, Fugu F2; 8, Medaka F1;9, PigB2; 10, Pig C1; 11, Pig F1; 12, Pig F2, 13, Dog F1;14, 100 bp marker.

To ensure that the DNA sequences we found were transcribed in the different species, we amplified cDNAs prepared from fresh tissue or cultured cell lines where these were available. Primers for the various species were based on our predicted mRNA sequences. PEDF sequences were amplified from dog, pig, chick, African clawed frog, zebrafish, fugu and medaka (Fig. 3). We also amplified African clawed frog serpin E2, fugu serpin F2, and pig serpins B2, Cl and F2. In each case we were able to amplify a band of the predicted size, confirming that the genes we detected in the genome databases are transcribed in adult animals.

### PEDF gene organization is conserved in vertebrates

**Figure 4:**
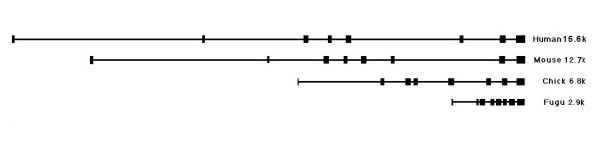
PEDF gene structure. Exon-intron arrangements of the PEDF gene in human, mouse, chick and fugu. The PEDF gene in each species consists of 8 exons and 7 introns. The considerable overall size difference of the PEDF gene among the species is almost entirely due to the variation in intron size. Black box shows exons and line shows introns.

The PEDF gene in all species examined contained 8 exons and 7 introns (Fig. 4), although there are variations in the lengths of these among the species as indicated in Table 2. Differences in exon sizes are much smaller than those observed for the introns, and one, exon 4, is identical in size across all phyla. The most significant changes in the structure of PEDF appear in fugu, with the largest exon size variations seen in the non-coding regions of exons 1, 2, and 8. In general the fish have smaller PEDF introns. The largest size variation in introns is seen in intron 1 with variations ranging from 683 bp in fugu to 27,524 bp in frog.

The complete genome sequences of human, mouse, chick, fugu, western clawed frog and zebrafish were used to examine the conservation of exon-intron splice junctions among the species. The 5' ag and 3' gt intron/exon splice junctions were conserved in all the species (Additional file 2). Statistically significant sequence similarities in the PEDF exons are seen between human and mouse, and between mammals and chick in exons 3–7, but no significant sequence homologies were detected in PEDF introns (data not shown). From this analysis we predict that a functional PEDF protein is produced in each of these species.

### A 200 bp proximal PEDF promoter region is conserved in vertebrates

**Figure 5:**
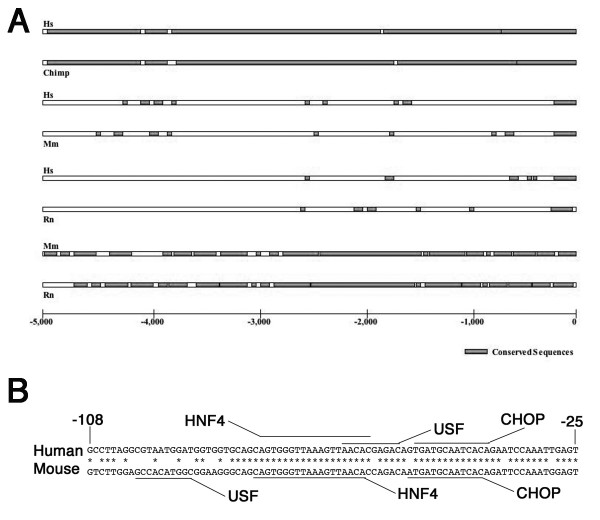
Promoter analysis of PEDF. **A. **Alignment of human, chimpanzee, mouse and rat PEDF promoter sequences. Alignment of the human and chimpanzee sequences show extensive homology, as do the mouse and rat sequences (homologous regions shown in grey). Very few conserved regions are seen when the human and mouse or human and rat sequences are aligned. The largest region of homology is the 200 bp upstream of the transcription start site. Right end of each line bar represents transcription initiation sites. **B. **Sequence comparison of 200 bp proximal region from human and mouse promoters. Star (*) shows conservation residuals. The location of each transcription factor binding site is indicated by lines.

We found no significant homologies between human and non-mammalian species when sequences 5' to the PEDF genes were compared. The more detailed analysis was, therefore, confined to mammals. In Figure 5A, we present pairwise comparisons of sequences that are 5 kb upstream of the PEDF transcription start site for human, chimpanzee, mouse, and rat. As expected, human and chimpanzee are almost identical. Similarly, mouse and rat show extensive regions of homology throughout the 5 kb region. On the other hand, pairwise comparisons of human with mouse and human with rat indicate that there are very limited regions of similarity in the PEDF promoter. The only substantial homologous region is the 200 bp immediately preceding the transcription start site. We scanned the first 200 bp of the PEDF promoter upstream of the transcription start site in human and mouse using TANSFACT DNA-binding matrix and identified putative DNA binding sites for the transcription factors HNF4, CHOP, and USF. These are clustered together within a 70 bp stretch of the promoter (Fig. 5B). Further upstream, there are isolated blocks of homology but these differ in position among the species. It is probable that the regulation of PEDF in vertebrate is controlled by this 200 bp promoter region.

### SINE and LINE transposable elements are present in the introns, exons, and promoter sequences of the mammalian PEDF gene

**Figure 6:**
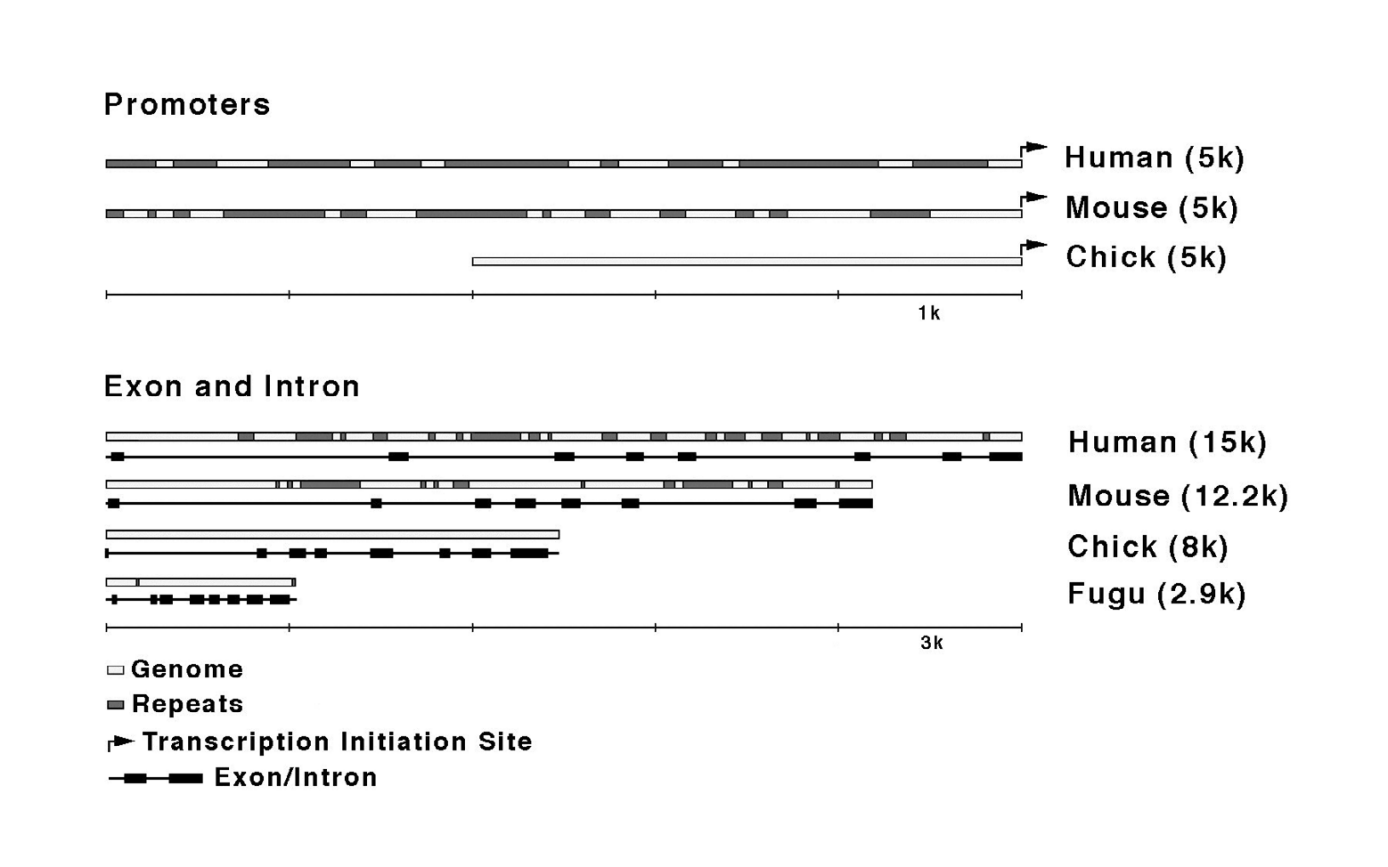
Repetitive elements in the PEDF promoter and gene. The promoter regions and genes are drawn to scale and the regions containing repetitive elements are shown in grey. A large portion of the 5 kb upstream region of human and mouse PEDF consists of repetitive elements. Repeats are localized to the promoter and introns of the gene. Open box, genome; black box, repeat regions; arrow, transcription initiation sites.

Alu's are mobile DNA elements inserted in genomic DNA shown to be rich in binding sites for transcription factors. A regulatory role for *Alu *sequences in differention and development has been established for several developmental genes where *Alu *was shown to act as an enhancer or silencer of gene expression [24]. *Alu *elements were first noted in the promoter region of the human PEDF gene [25]. A more extensive analysis of repetitive elements in the PEDF promoters and gene for four species is presented in Figure 6. In human PEDF, all the repeats are of the short interspersed nuclear elements (SINE) class, which are usually derived from tRNAs. Most of the PEDF SINE class repeats are of the *Alu *family but there are also a few Mir family repeats in the gene. In mouse, the elements were more varied. Many SINE class repeats were identified but there were also some long interspersed nuclear element (LINE) class repeats present. We have not been able to find any repetitive sequences in the chick PEDF gene or its promoter region using the available knowledge of repeat sequence structure in this species. We found only two short stretches of simple repeats in the fugu PEDF introns but have no information regarding repeats in the promoter because this region is less well-defined. In human and mouse, the repetitive elements in the promoters were extensive but sufficiently varied in length and position, as well as type, to substantially alter the overall structure of the 5' region. Within the PEDF gene, all the repetitive elements are located in the introns but again there were differences between human and mouse in length, position, and type of elements (Fig. 6).

### Conservation of order of genes flanking the PEDF sequence

**Figure 7:**
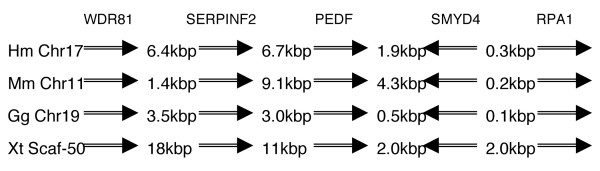
Orientation and distances separating PEDF and surrounding genes in human, mouse chick, and frog chromosome. Hm, Human; Mm, Mouse; Gg, Chick; Xt, African clawed frog; and bp, base pair.

Gene order conservation is a useful genomic measure for predicting functional interaction and relatedness among genes within a cluster. Selective processes are essential to preserve the organization of these clusters in closely related species. Conservation of the gene cluster is believed to be associated with the integrity of the cell and the functional relatedness of the genes comprising the cluster. In addition to the human and mouse, we obtained the sequence of the PEDF gene and surrounding region for chick, frog and several fish species from the genomic databases. Examination of an approximately 100 kb region of the human 17pl3.3 region shows that PEDF is immediately flanked by Serpin F2 (a2-antiplasmin) and WDR81 at the 5'end (Fig. 7). These three genes are separated by intergenic distances of 6.7 kb and 6.4 kb, respectively. At the 3' end, PEDF is flanked by SMYD4 and RPA1 at intergenic distances of 1.9 kb and 0.3 kb respectively. Mouse, chick and frog all showed the same arrangement of these five genes, although the intergenic distances varied substantially (Fig. 7). The results indicate that the genes immediately surrounding PEDF, are syntenic among species across three phyla.

**Figure 8:**
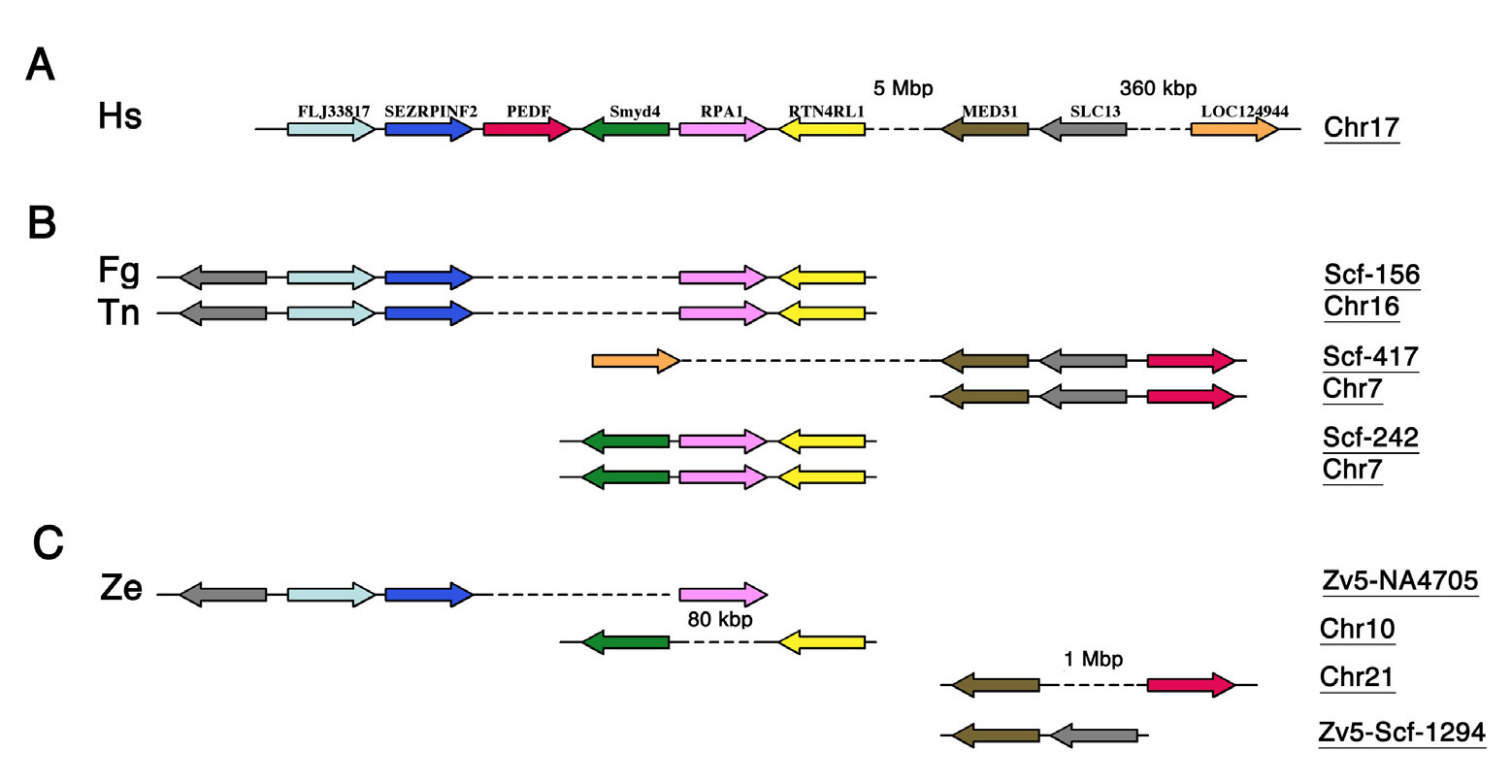
Genome structure comparison of PEDF and surrounding genes in mammals and fishes. **A. **The orientation and gene distances in human genome. **B and C. **The orientation and gene distances in fugu, tetraodon and zebrafish genomes. **B. **Fg, Fugu; Tn, Tetraodon; and **C. **Ze, zebrafish.

In the fish species, however, the organization of the regions around the PEDF gene was very different from that of human (Fig. 8A). In fugu and tetraodon (*Tetraodon nigroviridis*) the genes appear to be distributed among three chromosomes (Fig. 8B). On one chromosome FLJ33817 – SerpinF2 – (1.5 kb) – RPA1 – RTN4RL1 were identified. The PEDF gene is located on a different chromosome while Smyd4 and another RPA1 gene are mapped to a third chromosome. In zebrafish the genes are also arranged on 3 different chromosomes, although not identical to the other fish species (Fig. 8C).

### PEDF dN/dS ratio in mammals and fishes show that the gene is under strict purifying selection

The approach of non-synonymous/synonymous substitution rate ratio is widely used to detect positive selection (directional selection), neutral selection and negative selection (purifying selection) [26]. We have analyzed the dN/dS ratios between PEDF genes in three groups from different species: mammals (human, chimpanzee, pig, mouse and rat), fishes (fugu, tetraodon, and trout), and frogs (*X. laevis *and *X. tropicalis*). The dN/dS pairwise analyses show that the ratios between mammalian PEDFs are around 0.11 and the values between fish PEDFs are around 0.13, respectively (Table 3). No significant difference in dN/dS ratios was found between mammal and fish groups. The ratio between two frogs is 0.25. We also used the two-value model to test the difference between these two groups and the result was consistent with the ratios observed above, indicating that PEDF genes in mammals, fishes, and frogs are under strict purifying selection.

## Discussions and conclusions

PEDF is a unique member of the serpin supergene family. It functions as one of the most potent antiangiogenic factors so far described, is neuroprotective to cells in many parts of the nervous system, regulates the kinetics of exit from stem cell pools and inhibits growth of a number of tumors [8,9,27-30]. There is evidence that these diverse functions may be due to the activation of multiple signaling cascades in a cell type and microenvironment specific manner [15,31,32].

### PEDF is only found in vertebrates

We found a single PEDF gene in all vertebrate phyla examined from fish to mammals. We did not find PEDF homologues in fly, worm, or the sea squirt even though these species have many other easily detectable serpin genes. Recent phylogenetic analyses of genomic data indicate that tunicates, which comprise sea squirts, appendicularians and salps, are the closest living relatives of vertebrates [33]. Thus, it is likely that the PEDF gene evolved after the separation of the vertebrate lineage about 550 million years ago but before the separation of the major vertebrate phyla, at least 300 million years ago.

Several differences in structure and development between invertebrates and vertebrates may explain the need for regulatory factors such as PEDF. Most invertebrates develop largely in a strict lineage from a limited set of precursors. Vertebrates tend to rely more on generation of a pool of stem cells and from which differentiated progeny arise. PEDF plays an important role in this by regulating the size of the pool of stem cells [8]. As vertebrates evolved a vascular system, genes such as PEDF may have become essential to maintain the structural and functional integrity of the newly formed vessels. In human neovascular diseases, there is evidence that regulation of the balance between vascular quiescence and vascular proliferation is critical. Two factors implicated in maintaining this equilibrium are the antiangiogenic PEDF and the proangiogenic vascular endothelial growth factor (VEGF) [27,32]. VEGF homologues are found in insects where they function as regulators of hematopoetic cell production [34,35]. In early vascular evolution, the activity of VEGF could have been modified to promote vessel growth. At the same time, PEDF could have evolved to provide a counteracting influence essential to safeguard against abnormal vascular proliferation caused by VEGF. PEDF is also neuroprotective and it is possible that this function became important in vertebrates as they developed longer lifespans and a need to protect neurons against a variety of environmental insults.

### Differences in genes flanking PEDF between fish and other vertebrates

Hierarchical clustering of serpin genes confirms that the two most closely related to PEDF are serpin F2/a2-antiplasmin and serpin G1/C1 inhibitor. Serpin F2 is part of the same clade of serpins defined by the exon/intron organization [23,36]. Like serpin F2/a2-antiplasmin, serpin G1/C1 inhibitor is a classical protease inhibitor. This cluster of three genes is thought to have arisen from a common ancestor and is an example of ancestral serpins diverging into inhibitory and non-inhibitory subgroups. A common ancestral gene could have duplicated to give rise to the precursor of PEDF and the precursor of serpin G1/C1 inhibitor and serpin F2/a2-antiplasmin. The latter precursor then could have duplicated to allow the independent evolution of serpin F2/a2-antiplasmin and serpin G1/C1 inhibitor.

The PEDF region is syntenic in mammals, birds and amphibians, arguing for strong conservation of this gene cluster for a considerable evolutionary time. There are at least two possible mechanisms that maintain the integrity of this gene cluster. First, selective pressure on one gene in the region could create linkage disequilibrium that would effectively reduce structural alterations, which could disrupt the synteny. Second, the existence of rapidly evolving repetitive elements in this region may decrease the probability of chromosome rearrangements. Bony fish can be classified into two groups, the lobe-finned fish and the ray-finned fish. Lobe-finned fish are considered to be the probable ancestors of all land vertebrates, including mammals, reptiles, avians, and even amphibians. Ray-finned fish are the dominant group of vertebrates, with over 27,000 species ubiquitous throughout fresh water and marine environments. It has been proposed that a whole genome duplication occurred in the ray-finned fish lineage after its separation from lobe-finned fish about 450 Myr ago [37,38]. The fish species we examined all belong to the ray-finned fish group. The dispersal in fish of the cluster of genes around PEDF is probably a result of the whole genome duplication. Interestingly, each of the three fish species examined has retained only one copy of PEDF gene. One explanation of this is that PEDF expression is tightly regulated and that it will be very difficult to precisely control it by two separate promoters.

### PEDF could play similar roles in all vertebrates

In general, if one gene is under pure natural selection, which removes deleterious mutations from a population, then its dN/dS ratio will be less than 1.0. The smaller is this ratio means more critical functions the gene plays in the organisms. It stands in contrast to positive selection that means this gene is optimizing its function or even adopting a new function which will give the organism some advantage in the environment, and the dN/dS ratio will be greater than 1.0. In this study, the lower dN/dS ratio of mammals, fishes, and frogs analysis suggests that all PEDFs genes through vertebrates are under strict purifying selection, even though the dS and dN rates in fishes are obviously greater than in mammal, but the dN/dS ratios in these two groups are very close. These observations suggest that PEDF genes play critical roles in all vertebrates, and it is also very likely that they work in the same biological processes in the vertebrates.

### Conservation of PEDF gene structure through vertebrate species

All PEDF genes show structural conservation of 8 exons and 7 introns, with no evidence of exon shuffling or duplication. There are, however, some interesting size differences between these elements. The overall gene size varies from 2.9 kb in the condensed genome of fugu to 43.8 kb in frog. This variation is, in large part, due to differences in intron sizes. The greatest variability in size relationship was observed in frog, zebrafish, and fugu exons. Five of the exons, consisting entirely of coding sequence, were of identical lengths in human, mouse and chick. Only two exons of fugu PEDF were identical in size to those of the other species. The others had small reductions in length and result in a predicted protein that is only 18 amino acids shorter than human PEDF. The high conservation of the PEDF protein sequence in such distantly related species argues for conservation of the PEDF function throughout evolution. It is possible that the reduced intron sequences of fugu represent the minimum essential component of the gene structure that allows for functional activity of the PEDF gene.

The conservation of the PEDF gene does not extend into the promoter region. Analysis of approximately 5 kb of 5' DNA sequence revealed that only limited regions of homology exist even between mouse and human PEDF. The conserved proximal region does have promoter activity, as judged by transfection experiments [39]. A small group of conserved transcription factor binding sites was identified in the proximal promoter of PEDF. HNF4, hepatocyte nuclear factor 4, controls expression of specific genes in hepatocytes [40], and is involved with accumulation of hepatic glycogen stores, organization of the sinusoidal endothelium [41], and maturity onset diabetes of the young (MODY) [42]. CHOP/C/EBP homologous protein/DNA damage-inducible gene 153 serves as a dominant-negative inhibitor of the transcription factors C/EBP [43], controls endoplasmic reticulum (ER) stress-mediated apoptosis, and plays a central role in neuronal apoptosis during brain development [44]. USF is a ubiquitously expressed upstream stimulating factor and a member of the basic-Helix-Loop-Helix-Leucine Zipper transcription factor family with high affinity for cognate E-box regulatory elements. USF controls lipid and glucose metabolism and regulates genes involved in stress and immune responses [45]. Because of the strong conservation of these transcription factors in the mammalian PEDF promoter, we predict that the PEDF gene is under the control of these molecules in mammals and linked to their biological actions. PEDF levels increase in injury and decrease with senescence, elevated oxygen tensions, and during ischemia (reviewed in [9]), but the regulatory elements responsible for these changes are not yet known.

The varying numbers and positions of repetitive elements in the 5' region may cause a variable positioning of important regulatory regions such that they cannot be detected by routine homology searches. The *Alu *elements themselves may be another regulatory component of the human PEDF gene. *Alu *elements are a feature of primates only and related but different sequences are seen in the mouse PEDF promoters. *Alu *elements, once considered genomic junk, are now believed to contain genetic information important to primate evolution and gene regulation [46]. These elements are peppered throughout the human genome and contain a unique sequence that can be mutated at a single base to create a new splice site and give a different version of a protein [47]. Although *Alu *sequences are a significant proportion of the human PEDF promoter and introns, PEDF splice variants have not been identified. As with other serpins, one function of these repetitive elements may be to attach the PEDF gene to the nuclear matrix [48].

In summary, we have derived the structure and sequence of the PEDF gene from a range of vertebrate species. We have assembled PEDF sequences from 10 additional species and these can now be used to help us further understand the structural, regulatory, and functional conservation of this molecule. These new sequences also provide the necessary information to develop specific probes with which to study the expression and function of PEDF in these species. There is strong conservation in defined regions of the PEDF gene suggesting that these conserved sites are critical for function. Knowledge of the evolutionary dynamics of PEDF could be a valuable predictor of the function of this gene and provides the opportunity for understanding the genetics, regulation, and biologically essential domains of this molecule.

## Methods

### Data sources of cDNA and protein sequences

The human, mouse, and rat PEDF cDNA sequence were obtained from the UCSC Genome Bioinformatics Site [49]. A blastp search in a database of predicted genes was performed to detect the PEDF gene in fugu [50]. The non-redundant sequence database and EST database in NCBI were used to perform blastp searches to collect entire or partial cDNA sequences of PEDF and other serpin genes in species of chimpanzee, cow, pig, chick, trout, medaka, zebrafish, African clawed frog, western clawed frog, and dog [51].

### Data source of genome sequences

PEDF genomic sequences for mouse, rat, dog, western clawed frog, zebrafish and fugu were assembled using the BLAT tool in the UCSC Genome Bioinformatics Site [49]. Genomic information for other species was obtained by blastn search of Genome WGS (Whole Shotgun Sequence) database of each species [52].

### Phylogenetic tree analysis

Protein sequences of serpin genes (annotated or non-annotated) for species of sea squirt, fly and worm came from Ensembl database [53]. These sequences and other protein sequences of typical serpin genes in human and mouse were used to construct the phylogenetic tree of serpins. We also used protein sequences of all available PEDF and other serpins in vertebrate species to construct the phylogenetic tree.

All phylogeny trees were constructed using "MEGA", a molecular evolutionary genetics analysis software [54]. All protein sequences were aligned by clustal alignment function, then the bootstrap concensus tree was contructed by UPGMA method.

### Sequence similarity comparison between species

DNA Block Aligner was used to align DNA sequences in promoter region between species [55]. The subsequent model was a 3-state Hidden Markov Model (HMM).

Program 'fasta34' was used to pairwisely compare the exon sequences between two species [56]. Then the matrix in Table 1 was constructed by our python codes based on the result came from fasta program.

### Sequence repetitive analysis and other software

RepeatMasker [57] was used for repetitive element analyses on PEDF genomic sequences of various species. The EMBOSS package [58] was used for routine manipulation of sequences.

### Promoter analysis

The promoter sequences of PEDF gene from human and mouse genomes were obtained from UCSC Genome Bioinformatics website [49] and the possible transcript factor binding sites were examined in the -200 to +1 bp of each promoter sequence using the positional weighting matrices which are from TRANSFAC databases [59]. The footprints of sequence conservation between human and mouse promoters were generated by program Dna Block Aligner came from software package Wise 2.0 [55].

### Semi-quantitative PCR

Total RNA isolation was performed essentially as described before [60]. Primers designed for different species are shown in Additional file 3. cDNA was subjected to semiquantitative PCR using the PEDF primers listed below. PCR conditions used were: denaturing, 95°C for 1 min, annealing, 56°C for 1 min and elongation, 72°C for 1 min, for 30 cycles. PCR amplification products were electrophoresed on 4% agarose gels and bands were visualized by ethidium bromide staining. GAPDH or b-actin gene fragments were amplified as controls to determine PCR and cDNA loading efficiencies.

### dN/dS non-synonymous/synonymous substitution rate ratio analysis

CDS sequences for PEDF genes in species were obtained from NCBI. We assembled all CDS sequences and protein sequences of PEDF in three groups from different species: mammal, fish, and frog and stored into different files respectively. These CDS sequences were aligned by our home-made python program according to the alignment of their protein sequence. A program based on maximum likelihood algorithm was used to calculate the rate of nonsynonymous and synonymous substitution (program codeml, from Phylogenetic Analysis by Maximum Likelihood (PAML) website [26,61].

We run the program codeml in pairwise mode (runmode = -2) to calculate the dN/dS ratio between any two species in the same groups. The result was collected and formatted by our own python program and shown in Table 3. We also run the program codeml in two-value branch mode (runmode = 0, mode = 0; all mammals for one ratio value and all fishes for another ratio value) to calculate the average dN/dS ratio in each group from different species.

### GenBank submission

A total of 23 new serpin sequences, including 10 PEDF genes, were identified and submitted to NCBI GenBank as TPA (Third Party Annotation) under the accession numbers TPA: BK005442–BK005463 and BK005764. The accession numbers for individual serpin genes are A1, *X. tropicalis *cDNA [GenBank:BK005442]; A8, cow cDNA [GenBank:BK005443]; B1, pig cDNA [GenBank:BK005444]; C1, pig cDNA [GenBank:BK005445]; C1, *X. tropicalis *cDNA [GenBank:BK005446]; E2, *X. laevis *cDNA [GenBank:BK005447]; F2, *Fugu *cDNA [GenBank:BK005448]; F2, chicken cDNA [GenBank:BK005449]; F2, pig cDNA [GenBank:BK005450]; I1, cow cDNA [GenBank:BK005451]; I1, *X. laevis *cDNA [GenBank:BK005452]; F1, chicken cDNA [GenBank:BK005453]; F1, chimpanzee cDNA [GenBank:BK005454]; F1, *Fugu *cDNA [GenBankBK005455:]; F1. medaka cDNA [GenBank:BK005456], F1. pig cDNA [GenBank:BK005457], F1. trout cDNA [GenBank:BK005458], F1, *X laevis *cDNA [GenBank:BK005459]; F1, zebrafish cDNA [GenBank:BK005460]; E2, chicken cDNA [GenBank:BK005461]; E2, *X. tropicalis *cDNA [GenBank:BK005462]; F1, *X tropicalis *cDNA [GenBank:BK005463]; F1, dog cDNA [GenBank:BK005764].

### Accession numbers of invertebrate serpins

Sea squirt: [EMBL:ENSCINP00000001388, EMBL:ENSCINP00000006259, EMBL:ENSCINP00000015229, EMBL:ENSCINP00000018441, EMBL:ENSCINP00000024310, EMBL:ENSCINP00000026396, EMBL:ENSCINP00000029020]; Fly: [GenBank:NP_652024, GenBank:NP_609128, GenBank:NP_723300, GenBank:NP_723323, GenBank:NP_787995, GenBank:NP_609172, GenBank:NP_524957, GenBank:NP_609341, GenBank:NP_599148, GenBank:NP_524956, GenBank:NP_610188, GenBank:NP_524955, GenBank:NP_610244, GenBank:NP_610245, GenBank:NP_524958, GenBank:NP_610246, GenBank:NP_524805, GenBank:NP_001027395, GenBank:NP_610261, GenBank:NP_524851, GenBank:NP_610289, GenBank:NP_610633, GenBank:NP_610823, GenBank:NP_611190, GenBank:NP_524953, GenBank:NP_001015428, GenBank:Q9VVWl, GenBank:NP_730388, GenBank:NP_730512, GenBank:NP_649206, GenBank:NP_649207, GenBank:NP_650290, GenBank:NP_788678, GenBank:NP_650427, GenBank:NP_651818, GenBank:NP_524637]; and Worm: [GenBank:NP_503315, GenBank:NP_503318, GenBank:NP_503528, GenBank:NP_504889, GenBank:NP_504890, GenBank:NP_001023824, GenBank:NP_505108, GenBank:NP_507268, GenBank:NP_507269].

## Authors' contributions

XX carried out all computational experiments, analysis, statistical studies, and software development. SZ was responsible for the experimental design, conduct, biological experiments of the studies, and primary drafted the manuscript and figures. CB and JT were responsible for coordination of the studies and drafted the manuscript and figures. All authors read and approved the final manuscript.

## Abbreviations

PEDF: pigment epithelium derived factor; LINE: long interspersed nuclear element; SINE: short interspersed nuclear elements; VEGF: vascular endothelial growth factor;

## Supplementary Material

Additional File 1 Phylogenetic relationship of PEDF to other serpins. A total of 88 serpins with 14 PEDF sequences were used to construct this bootstrap consensus tree based on 500 replicates. The numbers on nodes are bootstrap values. Click here for file

Additional File 2 Intron-Exon junction sequences of the PEDF gene for human, mouse, chick, western clawed frog, zebrafish, and fugu. Intron sequences shown in lower case and exon sequences in upper case. Click here for file

Additional File 3 List of PCR primers for PEDF RT-PCR analysis from each species. Click here for file

## Figures and Tables

**Table 1:** Nucleotide comparison of PEDF exons across species

Species 1 Species 2	Human Mouse	Human Chick	Human Fugu	Mouse Chick	Mouse Fugu	Chick Fugu
Exon1 e-value	1.9e-19	0.6	0.8	7.8e-02	0.7	1.0
Exon1 length	116	55	46	55	46	46
Exon2 e-value	3.9e-23	l.0e-02	0.8	7.9e-03	0.8	0.8
Exon2 length	96	86	61	86	61	61
Exon3 e-value	1.9e-57	4.2e-37	4.3e-13	l.le-36	2.9e-ll	0.9
Exon3 length	215	199	196	199	196	196
Exon4 e-value	5.2e-42	4.4e-22	1.2e-06	1.7e-26	3.7e-10	8.0e-10
Exon4 length	168	156	156	156	156	156
Exon5 e-value	1.7e-53	5.5e-22	1.7e-04	2.5e-25	3.7e-04	4.5e-09
Exon5 length	220	204	198	204	198	198
Exon6 e-value	3.5e-39	4.4e-27	3.0e-05	4.3e-28	9.2e-06	2.5e-03
Exon6 length	152	143	140	143	140	140
Exon7 e-value	6.7e-67	1.2e-41	5.0e-11	2.2e-42	1.9e-02	2.8e-13
Exon7 length	227	211	211	211	211	211
Exon8 e-value	1.9e-73	7.9e-38	5.0e-07	2.9e-43	3.2e-07	1.6e-06
Exon8 length	392	380	239	380	239	239

e-value: e-value come from fasta program.

length: length of the shorter exon.

**Table 2:** Exon and intron sizes for the PEDF gene from human, dog, mouse, chick and fugu

	**exon 1**	**intron 1**	**exon 2**	**intron 2**	**exon 3**	**intron 3**	**exon 4**	**intron 4**	**exon 5**	**intron 5**	**exon 6**	**intron 6**	**exon 7**	**intron 7**	**exon 8**	**bp SAM**
**Human**	128	4789	102	2743	199	772	156	567	204	2868	143	1119	211	308	379	14688
**Dog**	92	3740	92	1641	199	633	156	793	204	1178	143	1440	211	466	391	11341
**Mouse**	108	4449	88	1430	199	449	156	400	204	821	143	2812	211	440	362	12272
**Chick**	54	2542	88	415	199	79	156	778	204	803	143	378	211	361	379	6790
**Xenopus**	65	27524	83	4509	178	754	156	3216	204	3316	143	1313	193	1760	443	43811
**Fugu**	46	683	61	77	196	297	156	110	198	81	140	165	211	162	239	2822
**Zebrafish**	42	1753	64	133	190	99	156	1877	204	244	140	7681	211	94	255	13039

**Table 3:** Pairwise dN/dS ratio of PEDF CDS

**A**	**B**	**N**	**S**	dN/dS*	**dN**	**dS**
Mammals						
Rn	Pt	900.8	263.2	0.1083+-0.01844	0.0859+-0.01073	0.7931+-0.09281
Rn	Mm	882.0	282.0	0.1457+-0.03619	0.0321+-0.00632	0.2201+-0.03283
Rn	Hs	901.0	263.0	0.1085+-0.01840	0.0871+-0.01082	0.8031+-0.09382
Rn	Pig	893.3	270.7	0.1156+-0.01934	0.0929+-0.01134	0.8042+-0.09272
Pig	Hs	903.3	260.7	0.1026+-0.02068	0.0489+-0.00785	0.4766+-0.05824
Pig	Pt	903.7	260.3	0.1030+-0.02096	0.0477+-0.00773	0.4627+-0.05689
Pig	Mm	906.5	257.5	0.0961+-0.01691	0.0741+-0.00984	0.7705+-0.08789
Hs	Mm	911.8	252.2	0.0811+-0.01453	0.0690+-0.00941	0.8507+-0.09881
Hs	Pt	898.8	265.2	0.1124+-0.07728	0.0034+-0.00200	0.0305+-0.01094
Mm	Pt	912.3	251.7	0.0818+-0.01472	0.0677+-0.00930	0.8277+-0.09584

Fishes						
Tn	Fr	865.8	298.2	0.1372+-0.02576	0.0634+-0.00920	0.4620+-0.05480
Dr	Tt	861.5	302.5	0.1500+-0.02600	0.2482+-0.02086	1.6549+-0.24513
Tt	Fr	874.0	290.0	0.1023+-0.02414	0.2369+-0.02044	2.3160+-0.49873
Tn	Tt	885.7	278.3	0.1136+-0.02492	0.2451+-0.02082	2.1570+-0.43219
Dr	Tn	853.0	311.0	0.0258+-0.01722	0.3228+-0.02646	12.4958+-8.25131 **
Dr	Fr	852.0	312.0	0.0298+-0.02112	0.3283+-0.02657	1.0154+-7.74113 **

Frogs						
Xt	X1	899.8	327.2	0.2486+-0.05337	0.0525+-0.00814	0.2111+-0.02992

Note: *. value following '+-' is the SE value

**. This record is not reliable because of the synonymous saturation.
