# Supplementary material for: Molecular phylogeny of the antiangiogenic and neurotrophic serpin, pigment epithelium derived factor in vertebrates
Source: BMC Genomics. 2006 Oct 4;7:248. doi: 10.1186/1471-2164-7-248 (PMC1609119; doi:10.1186/1471-2164-7-248)
Supplement: Additional File 2 — Intron-Exon junction sequences of the PEDF gene for human, mouse, chick, western clawed frog, zebrafish, and fugu. Intron sequences shown in lower case and exon sequences in upper case. [file 1471-2164-7-248-S2.doc]

Human Exon1 cgctttaagaaaggagtaGCTGTAATCTGAAGCCTG // GCTGCAGCGTATCCACA**Ggt**aaagcagctccctggc 128bp

Mouse Exon1 cactttaagaaaagagtaGCTGTAATCTGAAGCCTG // AAGCTGCCGCAACCACA**Ggt**aaggcagcgcctggtc 108bp

Chick Exon1 cgctgagcctgcactgtcCCCGGCTCACACTCACTG // GGCCGAGAGGAGCCCAA**Ggt**aaggggcagccgaggc 56bp

Xenop Exon1 cacattcccagagtcaccGCCCGGGGGAGGTACCCT // GCAATTACCCTTGCCAA**Ggt**atgagcagaacttctt 65bp

Zebra Exon1 acagttggcggaggcagcTGAGAACTGAAAATCTGC // CGACGGTAAAGAAAACA**Ggt**cagacgctttattaac 42bp

Fugu Exon1 cagctggagtgcctccagTCAGTTTACATAACGGCT // GTTTGTCCGCCTGGAAG**Ggt**ctgggcgtgtttaatg 46bp

Human Exon2 ctcgttcttttcttg**cag**GCCCCAGGATGCAGGCCC // GCCAGCCCCCCGGAGGA**Ggt**cagtaggcaggcgggg 102bp

Mouse Exon2 ctcttgtttctcttg**tag**TTCCGGGATGCAGGCCCT // GTCCCCAGCAGCTCTGA**Ggt**cagtagggtgggtagg 88bp

Chick Exon2 atccttcttactccg**tag**GTTCAGGCATGCAGATTC // AACTCACCTGCTGGGCA**Ggt**aagtagaagacagcag 88bp

Xenop Exon2 tatctttacttctta**cag**AACTGACAATGAAGATCT // CAGAATGCTGCAGATGA**Ggt**aaaaaaaaactattac 83bp

Zebra Exon2 tttgacctcattcat**cag**GATGAAGAAGATAGTTCT // CTCTCACATGCTCAGTT**Ggt**aagatctatcagttta 68bp

Fugu Exon2 tctgtgtttgtcctc**cag**GATGAAAGGAACTACTTT // AGATTCTGCCAAGCTCA**Ggt**aaatgtctgattttac 61bp

Human Exon3 cttcctgtctcctgc**cag**GGCTCCCCAGACCCCGAC // TCTCGGCCCTCTCGCTG**Ggt**gagtgctcagatgcag 199bp

Mouse Exon3 ccttgtattctaacc**cag**GGCTCCCCAGTCCCGGAC // TCTCTGCCCTTTCTCTG**Ggt**gagtgtcaactgaaga 199bp

Chick Exon3 gctaaatgtacttgc**tag**AACTCTCCAACCACTGAT // TTTCTGGTCTCTCACTC**Ggt**gagtccctgccccttt 199bp

Xenop Exon3 cttataatgttccta**tag**GTCCCTACAGAGGTAGAA // TGTCAAGTCTTTCCTTG**Ggt**gagtttgataaagatg 178bp

Zebra Exon3 ctttacacatttcta**cag**GCAGACACAACAGATGCA // TTACACAGCTATCAATG**Ggt**aaagctttgccattgg 190bp

Fugu Exon3 cttatgtctgtgcta**cag**TCAGAGACAGAGGCTGAG // TGACACAGCTCTCAATG**Ggt**gacaacacaaatcctg 196bp

Human Exon4 catccttgtctctgg**cag**GAGCGGAGCAGCGAACAG // GGATCGTCTTTGAGAAG**Agt**gagtcgcctttgcagc 156bp

Mouse Exon4 tccttttctctctgg**cag**GAGCTGAACATCGAACAG // GAATTGTGTTTGAGAGG**Agt**cagtagccccaccccc 156bp

Chick Exon4 tgtccttctcgttgg**cag**GGGCTGGAGAACGAACAG // GCATCATCGTGGAGAAA**Agt**aaaagtcctttccctc 156bp

Xenop Exon4 gaaaaaaaaagttca**tag**GGGGTGGACAAAGAACTG // GAATCATGCTGGAGAGA**Agt**aattagcattgttctc 156bp

Zebra Exon4 tttgtcatccatcaa**gag**CGTCTGAACGGGCCGAGA // GCATCCTTTTGGCGAGG**Agt**gagtgctcttcatttt 153bp

Fugu Exon4 ttttttcatgctacc**cag**GAGGATCTGAACATGCTC // GCCTCTACCTGTCACGT**Cgt**aggttcacactggagt 156bp

Human Exon5 tgctccgcctcttct**cag**AGCTGCGCATAAAATCCA // GTGTGGCGCACTTCAAG**Ggt**gagcgcgtctccaatt 204bp

Mouse Exon5 gatctgtttcctctc**aag**AACTTCGAGTCAAATCCA // GCGTGGCTTACTTCAAG**Ggt**gagggcttccccactt 204bp

Chick Exon5 catcttctagtttcc**cag**GACTGAGAGTGAAATCTA // GGGCTGCTTACTTCAAG**Ggt**aaggatgctgtgcact 204bp

Xenop Exon5 tctctctataatgaa**cag**GGCTCAGGCTACGGATGG // GAACTACTTACTTAAAA**Ggt**acaattaactggctat 204bp

Zebra Exon5 tcancctgtgtgttg**cag**ANCTTCGTCTAAGGCTGG // TGCCTATTTTAAGGGTA**Agt**taactctacttcttat 207bp

Fugu Exon5 ttgaggatgtgtctg**cag**GTCTTCGTCTGAAGCAGG // GTGCTGCCTACTTCAAA**Ggt**aggtcagattccccga 198bp

Human Exon6 tcatacacttctttc**cag**GGCAGTGGGTAACAAAGT // TCAGATCTCAGCTGCAA**Ggt**ctgtagggataggggc 143bp

Mouse Exon6 ccatctgtttccttc**cag**GGCAGTGGGTAACCAAGT // TCTGATCTCAACTGCAA**Ggt**ctgtagggatggaggt 143bp

Chick Exon6 tatcttttaaaaaaa**cag**GGACTTGGAAAACCAAGT // TCAGAGCTCAACTGCAA**Ggt**cagacagcaaatattg 143bp

Xenop Exon6 tttctgaatttataa**cag**GCCAGTGNGCGTACAAAT // TCTGATTTTNAACTGCA**Ggt**gtgaaagctttcttct 143bp

Zebra Exon6 tgcattgactttttg**cag**GTAAATGGATAACTAGAT // TCTGACCTCGGCTGCAC**Ggt**accagaatctgttgta 140bp

Fugu Exon6 gttgtcgtcatcaaa**cag**GGAAGTGGGTGACGCGGT // TCCGATTTGAAGTGCAC**Ggt**aactgtatccgtcagt 140bp

Human Exon7 ctttctcacttgtct**cag**ATTGCCCAGCTGCCCTTG // AGTCCCTGCAGGAGATG**Agt**atgtctgaagaccctt 211bp

Mouse Exon7 gcatctctgttgggt**tag**ATTGCCCAGCTGCCCTTG // AGTCTCTGCAGGACATG**Agt**atgtttgcaaggccac 211bp

Chick Exon7 ttataactggtcttc**cag**ATTGCCCAGCTGCCACTG // ACACTGTAAAGGAGACA**Agt**atgtacatcacattct 211bp

Xenop Exon7 acacatttttcctta**cag**ATTGTTCAGCTTCCTCTC // ATATGAAGCTGACCTTA**Aga**gcctgcaggaatcagt 193bp

Zebra Exon7 actgttaccctgtta**cag**ATAGCCCAGGTTCCTATG // CCTCCCTCTCTGACCTG**Ggt**gagacaaaaacaacat 211bp

Fugu Exon7 gcccactgtctcctc**cag**ATCGCTCAGATCCCGATG // CACTGCTGGGGGACCTA**Ggt**gagtggaagacctcca 211bp

Human Exon8 ctctctccatctcta**cag**AGCTGCAATCCTTGTTTG // AAAGAGCTTTATCCCTAActtctgttacttcgttcc 379bp

Mouse Exon8 atctctgaatctcca**cag**AGCTACAGTCGTTGTTTG // ATAAAAGAGCTTTTCCTTaatgtttgcttcttcctt 362bp

Chick Exon8 tttctttctttcctc**cag**GGCTCCAGTCACTTTTCA // CCTTATGCCTTTATGCTTgaataaaagagcttttgc 379bp

Xenop Exon8 attgttaattattta**cag**AGCTCCAATCCCTTTTCG // TTAAATCATTTTCCAGAAgacaattttcccacagac 443bp

Zebra Exon8 tttgttctcccgttt**cag**GTCTATCTGAATGGTTAG // CTCTAAAGGGTATGAGACaaaaacccccatcccctc 269bp

Fugu Exon8 tctctacctccattg**cag**GTCTTTCTGATTGGCTAT // GTGGTCAACCCTACTGGCctaacaatttaacacatg 239bp
