# Supplementary material for: Molecular phylogeny of the antiangiogenic and neurotrophic serpin, pigment epithelium derived factor in vertebrates
Source: BMC Genomics. 2006 Oct 4;7:248. doi: 10.1186/1471-2164-7-248 (PMC1609119; doi:10.1186/1471-2164-7-248)
Supplement: Additional File 3 — List of PCR primers for PEDF RT-PCR analysis from each species. [file 1471-2164-7-248-S3.doc]

Table 4. Primers used to detect novel serpin expression. All primer sequences were derived from genomic DNA information. All primer pairs are derived from different exons to ensure amplification from mature RNA.

Serpin Direction Sequence (5’ to 3’) Size

| SerpinB2.Pig | Fwd | GAAATTCCCATTCGGCTACA | 500 |
| --- | --- | --- | --- |
|  | Rev | TGTTGGAGGAGGGATTATGC |  |
| SerpinC1.Pig | Fwd | CATTTACCGTTCCCCAGAGA | 500 |
|  | Rev | GGTTGATGATCCCTCTGGAC |  |
| SerpinE2.Xl | Fwd | AGTGGAGGGCTCCTTTGTCT | 504 |
|  | Rev | TGAACCCGCTTAGGACTCAT |  |
| SerpinF1.Chick | Fwd | TTCAGCCTGGCTACTGCTCT | 500 |
|  | Rev | GCTTTGGGGTCTGACATCAT |  |
| SerpinF1.Dog | Fwd | ATGTGCTGCTGTCACCACTC | 506 |
|  | Rev | TGACATCATGGGGACTTTCA |  |
| SerpinF1.Fugu | Fwd | GCTACAACCTCTTCCGTGCT | 500 |
|  | Rev | AAAGGTGTCCATTGCTCCAC |  |
| SerpinF1.Medaka | Fwd | GGAGCTGTTCACAACACCAA | 508 |
|  | Rev | AAAGTAGGAGGCGCTCACAG |  |
| SerpinF1.Pig | Fwd | GGAAAAGTCATACGGGACCA | 510 |
|  | Rev | GACTTCGTGAGTTCGCCTTC |  |
| SerpinF1.Xl | Fwd | GCGAATCATGCTGGAGAGA | 500 |
|  | Rev | TCTGCAGTGCCTGGTCTATG |  |
| SerpinF1.Zebra | Fwd | ATCAATGGGAGCGTCTGAAC | 503 |
|  | Rev | AGCCGAGGTCAGAGTCGATA |  |
| SerpinF2.Fugu | Fwd | CCCCTAAATGTGCTCGTCAT | 503 |
|  | Rev | GCACCTTCCTCGTTGATCTC |  |
| SerpinF2.Pig | Fwd | GCAGAAAGGATTTCCCATCA | 513 |
|  | Rev | AGCTTAGGCAGCTGGACCTT |  |
